# Environment-sensitive motion modelling in healthcare with synthetic retargeting

**DOI:** 10.1177/20552076261418835

**Published:** 2026-01-30

**Authors:** Xiaodong Guan, Robert Gray, Yee-Haur Mah, Aryan Esfandiari, Jorge Cardoso, Parashkev Nachev

**Affiliations:** 1 Queen Square Institute of Neurology, 4919 University College London, London, UK; 2 4616 School of Biomedical Engineering and Imaging Sciences, King's College London, London, UK; 3 King's College Hospital NHS Foundation Trust, London, UK

**Keywords:** Synthetic data, domain adaptation, computer vision in healthcare, human pose estimation, human movement modelling

## Abstract

**Objective:**

To address the critical data scarcity and privacy constraints that limit video-based motor behaviour assessment in clinical settings through a synthetic data generation framework, enabling robust human detection with high fidelity across challenging scenarios.

**Methods:**

We employed synthetic data generation tailored to specific environments, implementing a novel synthetic retargeting approach based on procedural image synthesis. This method addresses the critical obstacles of limited training data in clinical settings due to privacy concerns, constrained views, occlusions, and uncontrolled environmental characteristics.

**Results:**

Our synthetic retargeting approach yielded substantial and statistically significant performance improvements in human detection under real-world clinical data regimes. Evaluated across two clinical scenarios, the method improved existing models’ performance (human detection score) by up to 19.4% in the more challenging scenario and up to 9.8% in the less challenging scenario (both with *p* < 0.001), demonstrating both high fidelity and robustness against challenging environments.

**Conclusion:**

Synthetic retargeting provides an efficient and effective solution for adapting pre-trained human detection models to specific clinical deployment scenarios by generating scenario-tailored synthetic data, circumventing the privacy and logistical constraints that limit real data collection in healthcare settings. This approach enables robust video-based motor behaviour quantification with significant implications for both clinical management and research.

## Introduction

**Figure 1.**
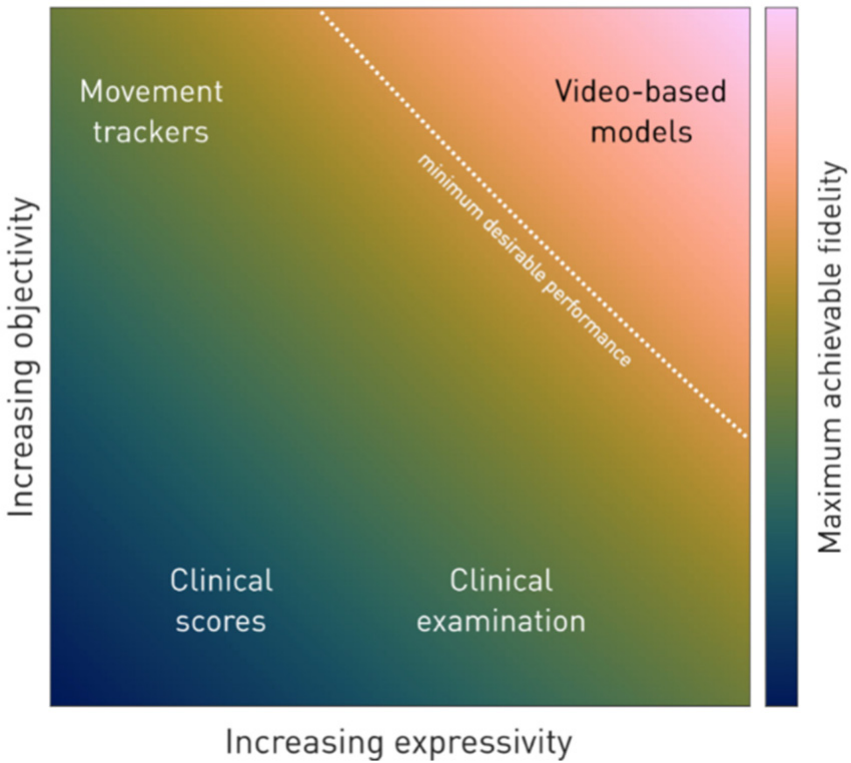
Schematised relation between the objectivity and expressivity of descriptions of motor behaviour, and their expected fidelity in downstream clinical tasks. Current methods lack objectivity or expressivity or both; video-based methods offer currently the only real-world deployable solution, but are challenging to design and implement.

Optimal neurological management often depends on faithful characterisation of a patient's motor behaviour. The underlying tasks range across triage, diagnosis, phenotyping, prognosis, treatment, dynamic monitoring, and closed loop interventions, invoked in both direct clinical care and the neuroscientific research that informs it. The complexity of motor behaviour renders such characterisation extraordinarily challenging, especially under the practical constraints of real-world observational settings. Theoretically the ideal approach – realisable only by automated capture (or accurate inference) of every joint location with high temporal resolution and breadth, sensitive to the environmental context – combines high objectivity with great descriptive richness (illustrated in Figure 1). Current practice sacrifices either or both of these desiderata, employing either objective, high-frequency sampling of single anatomical locations (e.g. wrist-worn trackers), or subjective, low-frequency sampling of unstructured (e.g. clinical examination) or structured (e.g. reductive classifications and summary scores) clinical descriptions.^1–3^ The former is limited by the expressivity of single/few joint locations; the latter by the inter-rater variability, expertise sensitivity, and resource cost/scalability of any subjective measure. That no current method is adequate is indicated by the comparatively low predictability of motor behaviour – in either clinical or pathological contexts – despite its obviously structured form.

Realising the ideal approach is hindered by the nature of real-world clinical settings. Richly featured marker-based methods of the kind used in the motion-capture industry place impossible demands on both patient and local environment. Markerless, deep learning-based, video capture methods are the only plausible alternative, but they require machine vision models with sufficient expressivity to capture the wide diversity of possible appearances, under the minimally controllable setting of clinical practice, including variable lighting, image noise, and object occlusion.^4–7^ Crucially, such models inevitably rely on large-scale, unselected, fully inclusive training data, including densely annotated joint-position labels, whose acquisition is powerfully inhibited by privacy and resource considerations universal across healthcare. This places us in a Catch 22: to develop sufficiently performant machine vision models, we need privacy-preserving, automatically annotated data that cannot be acquired without them.

**Figure 2.**
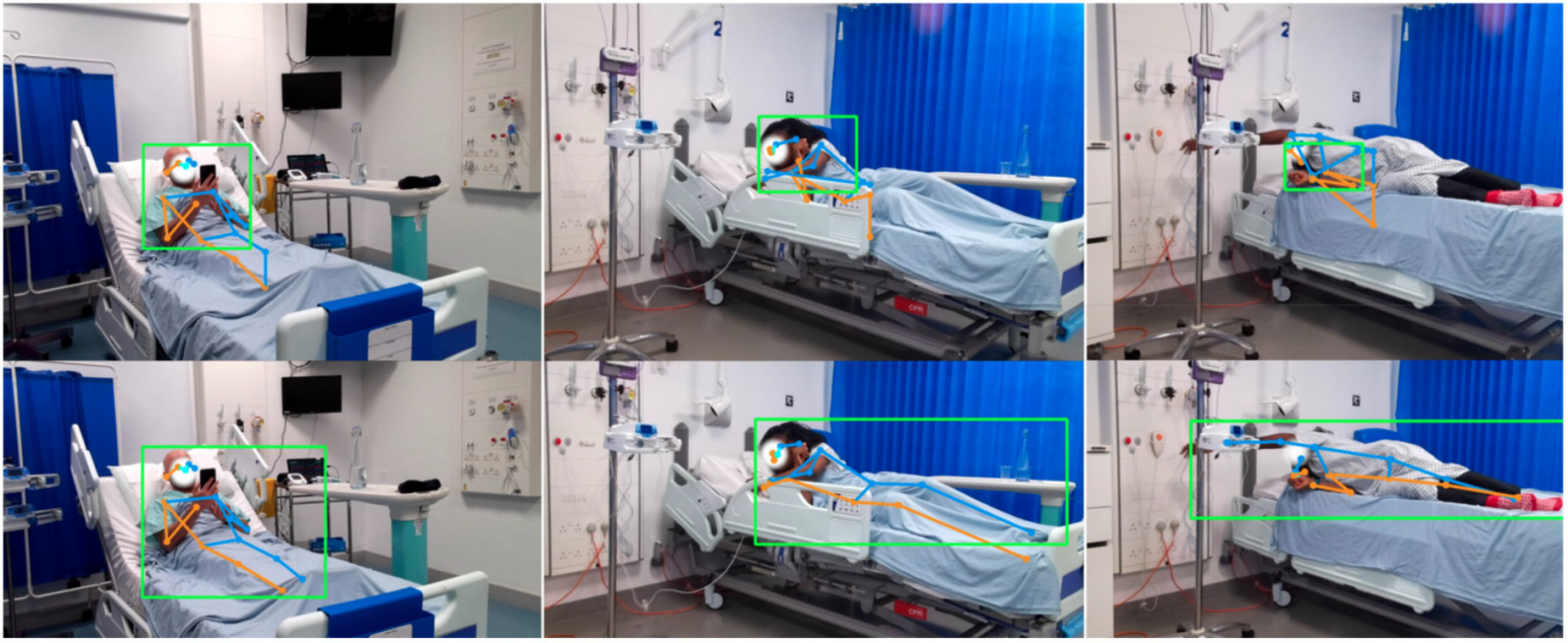
Illustration of human detection and pose estimation in a clinical context. Green boxes indicate the predicted positions of the human by a YOLOv6-L model before (top row) and after (bottom row) being trained on our synthetic datasets for one epoch. The blue and orange lines indicate left and right limbs predicted by a pre-trained ViTPose-huge model. The improved precision on human bounding box significantly increases the pose estimation performance.

The difficulty is easily demonstrated. Currently the best performing joint annotation (pose estimation) models approach the task hierarchically, beginning with human detection, where an object detection model computes bounding boxes for human instances, and proceeding to single-person pose estimation, where a pose estimation model locates body joints within the computed bounding boxes.^8,9^ The initial human detection is highly sensitive to the representation of both foreground and background visual characteristics in the training data; healthcare settings are both too rarely imaged and insufficiently similar to others for off-the-shelf pre-trained models to perform well (top panels in Figure 2). Crucially, the more unusual the clinical scenario, the less likely it is to have sufficient support in the training data, resulting not only in poor performance but clinically-relevant inequity in fidelity.

To quantify these concepts precisely, in this work, fidelity refers to detection accuracy quantified through average precision (AP), average recall (AR), and our per-person scoring system based on intersection area divided by union area (IoU) (section ‘Statistical analysis’). Equity refers to consistent performance across diverse deployment scenarios characterised by varying environmental constraints, viewing angles, and clinical contexts – operationalised through cross-scenario performance analysis and statistical significance testing demonstrating reduced performance disparity after synthetic retargeting.

**Figure 3.**
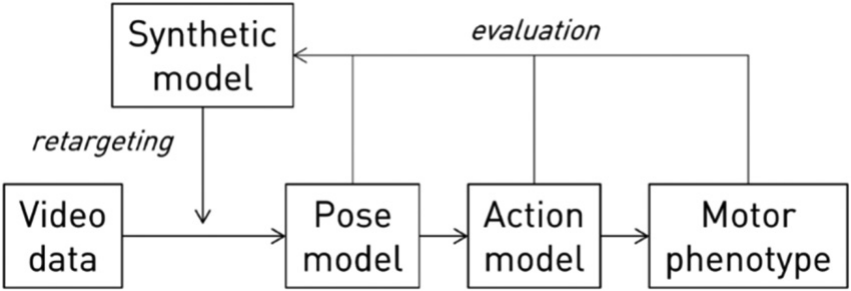
Outline of the proposed framework for enabling automated expressive characterisation of motor behaviour with the aid of synthetic data. A synthetic model is used to generate data crafted to optimise the performance of object detection and pose estimation models within a specific environment without the need to collect real data. Prior knowledge and evaluation of downstream performance can guide the synthetic generation.

Here we propose a comprehensive solution to this impasse based on finely controllable, targeted synthetic image generation. The central idea (as shown in Figure 3) is to train new, or tune existing, video-based joint annotation machine vision models with synthetic data specifically crafted to enable performance in real-world clinical settings. The synthetic data combines richly specifiable images of patients in clinical environments optimised for the target – potentially at the individual level – with ground-truth joint annotations, under such body morphology, pose, viewpoint, image noise, and occlusion parameters as the circumstances demand. The absence of available labelled data is thus remedied by synthetic data of the necessary kind. Model validation (and potentially fine tuning) naturally still requires feedback from real-world data, but the scale requirement for this is considerably lower and may include sparse, more easily derived annotations such as whole-body action labels. Crucially, the characteristics of the synthetic data may be both efficiently specified in advance and learnt end-to-end. The choice of both synthetic and annotation models is deliberately left open, to allow for the rapid innovation in both generative and discriminative model architectures that characterises the field of artificial intelligence.

The idea of using synthetic images to train computer vision models is well established.^10^ Training on synthetic data, alone or combined with real data, has yielded persuasive results, demonstrating that the right neural network architectures have the capacity to acquire knowledge from synthetic data and effectively transfer it to real-world scenarios. Generating suitable synthetic data requires a mechanism for specifying three-dimensional (3D) human figures of varying number, appearance, pose, and presentation, and embedding them across a controllable variety of backgrounds, so that a ground truth for the pose characteristics can be combined with a diversity of visual appearances.^10–14^ An efficient approach is *procedural synthesis* exploiting the 3D image rendering capabilities of naturalistic game engines such as Unity3D and Unreal or modelling software such as Blender.^15–17^ The challenge is to enable fully automated image generation that provides the wide variety of both foreground and background appearances manifest in real-world scenarios, with sufficient control to allow effective targeting to specific contexts. Researchers have exploited video games with diverse characters and automated baselines such as SMPL and SMPL-X to avoid the expensive manual design of virtual human figures while rendering realistic human mesh objects in diverse postures with acceptable fidelity.^11,18,19^ Despite the intense pursuit of high-quality synthesis through augmentation techniques and generative methods, and the strikingly realistic images generated by diffusion models, there remains a ‘reality gap’ between real and synthetic data an optimal approach must bridge.^20–25^

The reality gap does not prevent deep learning models from learning, but requires an adroit training strategy to minimise the risk of overfitting to the peculiarities of synthetic images.^26^ One approach is to mix synthetic and real-world data in training; another is to combine procedural, game engine-derived images with samples from diffusion models. Cai et al.^11^ collected data from a video game specifically for outdoor scenes and fine-tuned models to improve their performance in outdoor environments.^10,12,27,28^ Patel et al.,^13^ Black et al.,^14^ and Marcard et al.^29^ manually placed mesh objects, adjusted shadows, and employed artists to design human figures to increase image fidelity. Although these carefully curated synthetic datasets can closely approximate real-world appearances, the considerable resource cost limits application to highly diverse clinical contexts, and renders rapid, targeted adaptation infeasible.

In this paper, we propose a comprehensive, lightweight, low-resource pipeline for such targeted synthesis, and evaluate its utility in improving the performance of deep learning-based human detection models, with benefits for downstream pose estimation. We combine human body mesh generation models from SMPL-X with Unity3D rendering of diverse scenes, spanning a wide range of modifiable appearances, with special attention to challenging, non-canonical viewpoints.^15,19^ We tune state-of-the-art object detection models on our synthetic data, evaluating their performance on real video data derived from a controllable physical replication of a real-world clinical environment.^30–33^ We quantify the effect of synthetic retargeting compared with conventional augmentation, and establish the specificity of targeted manipulation through a comparison of tuning with canonical versus non-canonical viewpoint synthetic data.

## Materials and methods

This was a methodological development and validation study aimed at creating and evaluating a synthetic data generation framework for pose estimation in clinical settings. The study was conducted collaboratively across University College London, King's College London, and King's College Hospital in London, United Kingdom. The study timeline extended from 2021 to 2025, comprising three sequential phases: (1) synthetic data generation framework development (2021–2023), (2) clinical video data collection from patient populations (2023–2024), and (3) data pre-processing, model training, and performance validation (2024–2025).

Our investigation requires an image synthesis framework, a validation framework, and downstream pose analysis model training and evaluation. Here we focus on two key downstream tasks – human detection and two-dimensional (2D) pose estimation – to quantify the potential value of applying the proposed approach.

### Image synthesis framework

Our objective is to enable the synthesis of realistic scenes spanning a wide range of potential appearances, with full control over their characteristics so that those associated with underperformance of a chosen pose analysis model can be specifically amplified in training. The breadth of potential appearances makes this a very challenging task.

**Figure 4.**
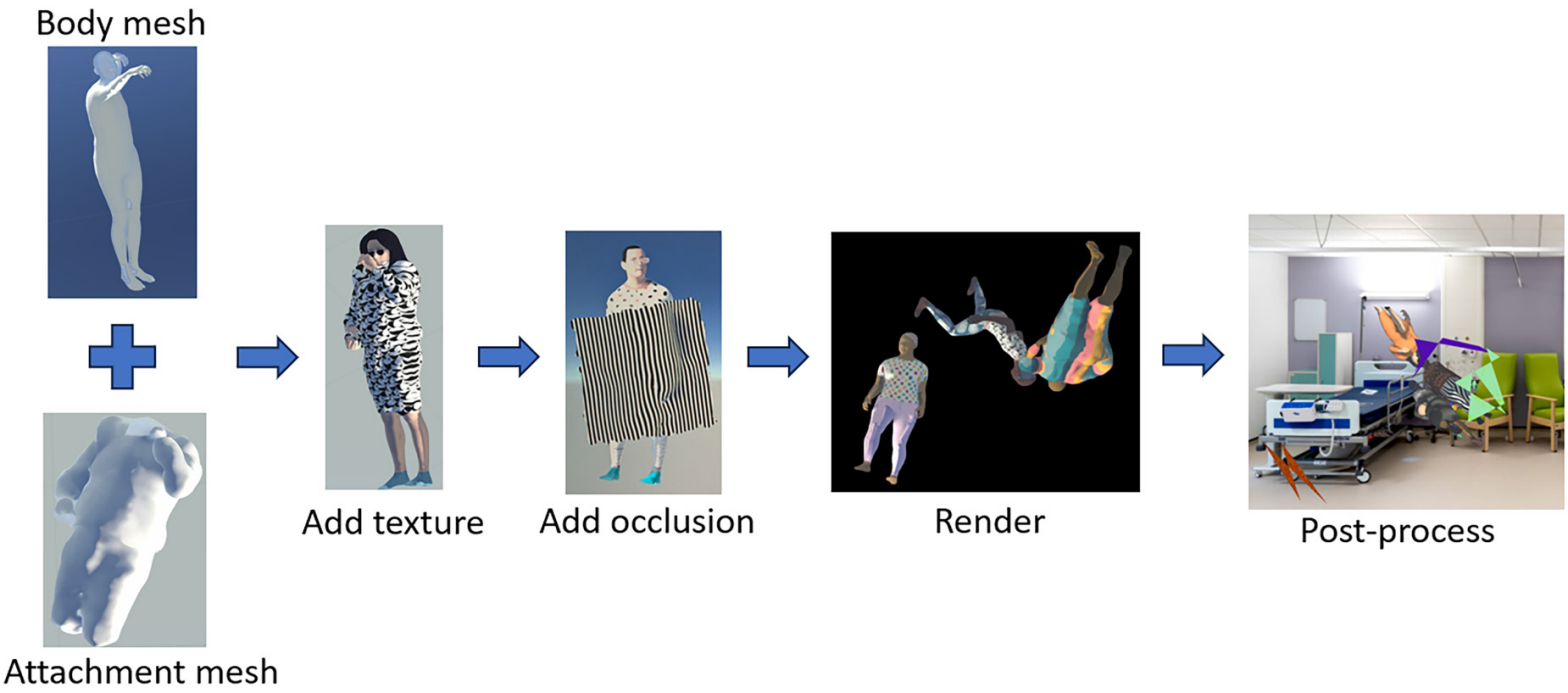
Flow chart of the image synthesis process.

Following the flowchart shown in Figure 4, the first step is to create a set of human body objects disposed in realistic poses. Synthetic human body mesh objects were generated using SMPL-X with sampled poses from AMASS, an archive including 24 human scan and motion capture datasets containing 17,916 motion sequences, with a total length exceeding 62 hours across over 500 participants.^19,34^ SMPL-X (Expressive Body Model) is a parametric statistical model representing 3D human bodies through shape parameters (body proportions), pose parameters (joint angles), and expression parameters (face and hands). The model decomposes human appearance into controllable components, enabling procedural generation of diverse synthetic instances by sampling from parameter distributions. This parameterisation allows systematic variation of body types, poses, and appearances while maintaining anatomical plausibility. The poses are first converted to joint-wise rotation in the axis-angle format according to the kinematic tree defined in AMASS and SMPL-X. Compared with Cartesian format, the converted representation excludes the influence of translation and can be easily applied on human mesh objects with diverse body shapes.^19,34^

The second step is to introduce realistic variation in morphologies of body and simulated clothing. The body shape is here determined by the SMPL-X mesh's betas term consisting of 10 values sampled between −1.0 and 1.0, adhering to a normal distribution with an average of 0.^19^ The randomly generated body shape descriptors and sampled poses were fed to SMPL-X to create plausible human body mesh objects.^19^

**Figure 5.**
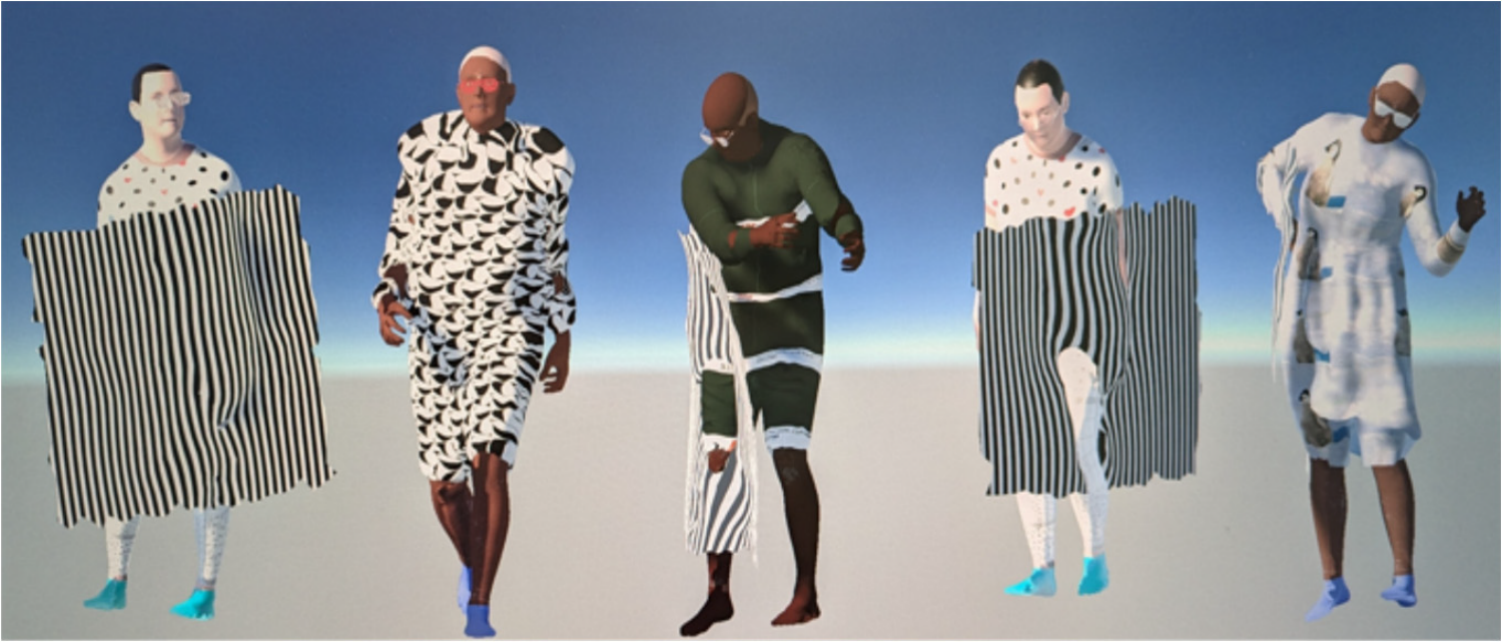
Examples of rendered human figures with attachments.

The third step is to apply realistic textures to the figures, simulating wide variation in skin tone and clothing. Skin colours were randomly sampled from an RGB skin-tone palette, and randomly applied clothing samples were obtained from copyright-free images harvested from the internet with appropriate keywords. Specifically, when rendering the appearance of each figure, we randomly selected whether to (1) directly use the mesh texture map to create a painted ‘naked’ body or (2) create random clothing mesh objects corresponding to the actions of the synthetic humans for more realistic deformation.^35^ Hair and eyewear were also randomly manipulated for further diversity of appearances (Figure 5).

The fourth step is to introduce occlusions that naturally limit the visibility of human figures in real-world hospital environments. We generated synthetic blankets commonly found in hospitals, covering the patients and thus creating additional challenges for deep learning models. The blanket mesh objects were created by adapting a planar mesh to conform to random parts of the human mesh surface, with added random noise and smoothing to provide nuanced details for neural networks (Figure 5) rather than introducing counterproductive complexity. This approach significantly reduces manual labour compared to other methods such as those proposed by Varol et al.,^10^ Cai et al.,^11^ and Black et al.^14^

The fifth step was to render the scene using a game engine such as Unity3D or other renderers (in this work we employed Unity3D for rendering, but the pipeline is renderer-agnostic and transferable to alternative platforms). We then specified the camera viewpoints and lighting configurations to render the complete scene. For each rendered scene, five directional lights were strategically placed around the human body mesh – specifically from the front, back, left, right, and above – each oriented directly towards the mesh. The intensity of each directional light was independently sampled from a uniform distribution ranging from 0.3 to 3.0, introducing a diverse set of lighting conditions.

A virtual camera was configured to capture the scene. Initially oriented towards the human body mesh, the camera was positioned at a distance uniformly sampled from the range [0.3,0.6] units in Unity3D.^15^ To ensure diverse yet realistic viewing angles, the camera's position around the mesh was randomised by independently sampling vertical angles within [−54∘,18∘] and horizontal angles within distinct ranges to simulate different scenarios (detailed in section ‘Validation framework’). The carefully restricted angular range prevented excessively high or low viewpoints, thus minimising undesirable perspectives and maximising the proportion of effective training samples. These simulated angle ranges are also intentionally broader than those typically encountered in real hospital settings, aiming to not only approximate real-world conditions but also account for rare scenarios by slightly expanding the distribution of viewpoints. Additionally, the camera is randomly rotated around its optical axis (*z*-axis), with the rotation angle uniformly sampled from the range of [−115∘,−30∘] , to simulate the posture of patients lying flat or reclining in bed – a posture commonly observed in hospital wards. The asymmetry in this rotation range accounted for differences between the anatomical reference origin of the human mesh (located at the pelvis) and the Unity3D world coordinate system, resulting in anatomically plausible and visually balanced renderings. Each scene configuration was rendered four times, resulting in 10,800 images per scenario. This number was chosen to plausibly balance image diversity and training capability but can naturally be expanded as far as capacity allows.

**Figure 6.**
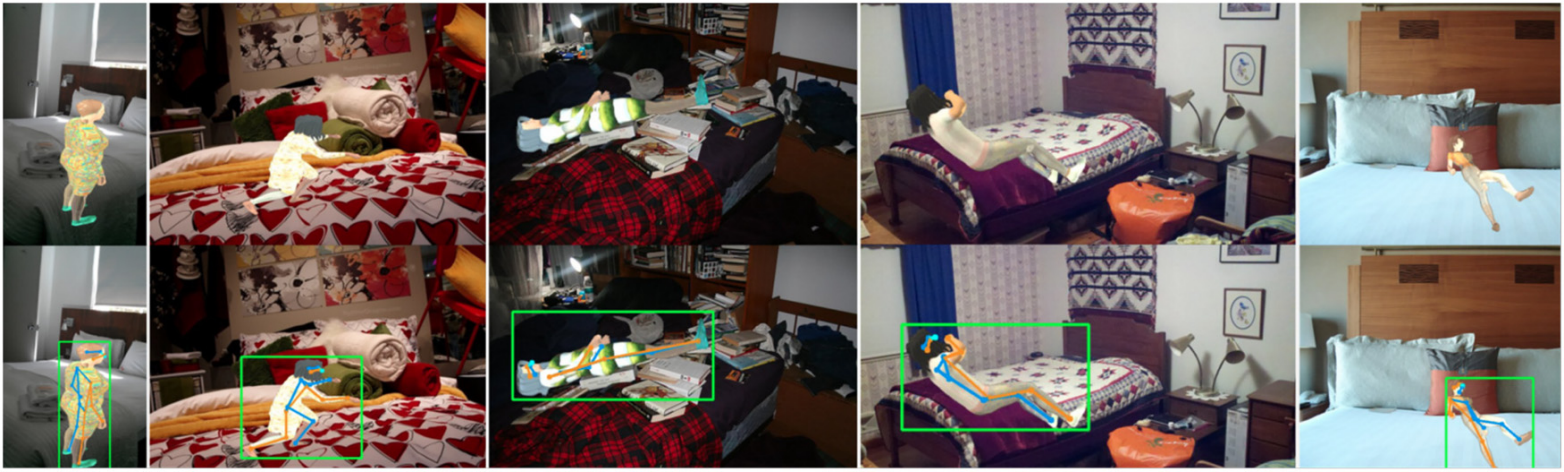
Examples of synthetic images with modified background images sampled from the MS-COCO dataset (images sourced from Flickr, licensed under CC BY 2.0).^36^ Green lines indicate ground-truth bounding boxes while blue and orange lines indicate separately left and right limbs.

The final step is to provide a variety of backgrounds in which figures are embedded to create a realistic scene. Here we used 280 harvested indoor bed-containing images as backgrounds. Within each scene, one human mesh object was rendered under varied lighting conditions, with transparent backgrounds (randomly selected), allowing seamless integration into diverse background images. When merging the human instances with background images, a pre-trained YOLOv6 model was employed to locate the positions of beds in the images so the human instances could be randomly placed near or on the beds (Figure 6).^31^

The ground-truth 2D and 3D positions of all human body joints and bounding boxes were tracked throughout the process and saved as annotation files for training, validation, and testing.

### Validation framework

Validation requires a real-world simulation environment where we can replicate a wide range of clinical scenarios and record them on video with control over the recording parameters such as camera viewpoint.

We collected video data within the Motion Analysis in Simulated Clinical Environments project, registered and granted ethical clearance with King's College Hospital (reference number: MRPP-22/23-34811).^33^ A total of 21 participants were involved, with 14 participants assuming the patient role, seven participants assuming clinician roles. The mean age of the patient-role participants was 41.4 years (SD 17.2 years, range 26–72 years), and the mean age for all participants was 38.0 years (SD 14.8 years, range 23–72 years). There were thirteen females and eight males in total. Classified according to UK 2021 census groupings, the participants encompassed Asian or Asian British; Black, Black British, Caribbean or African; White; and other ethnic groups.

**Figure 7.**
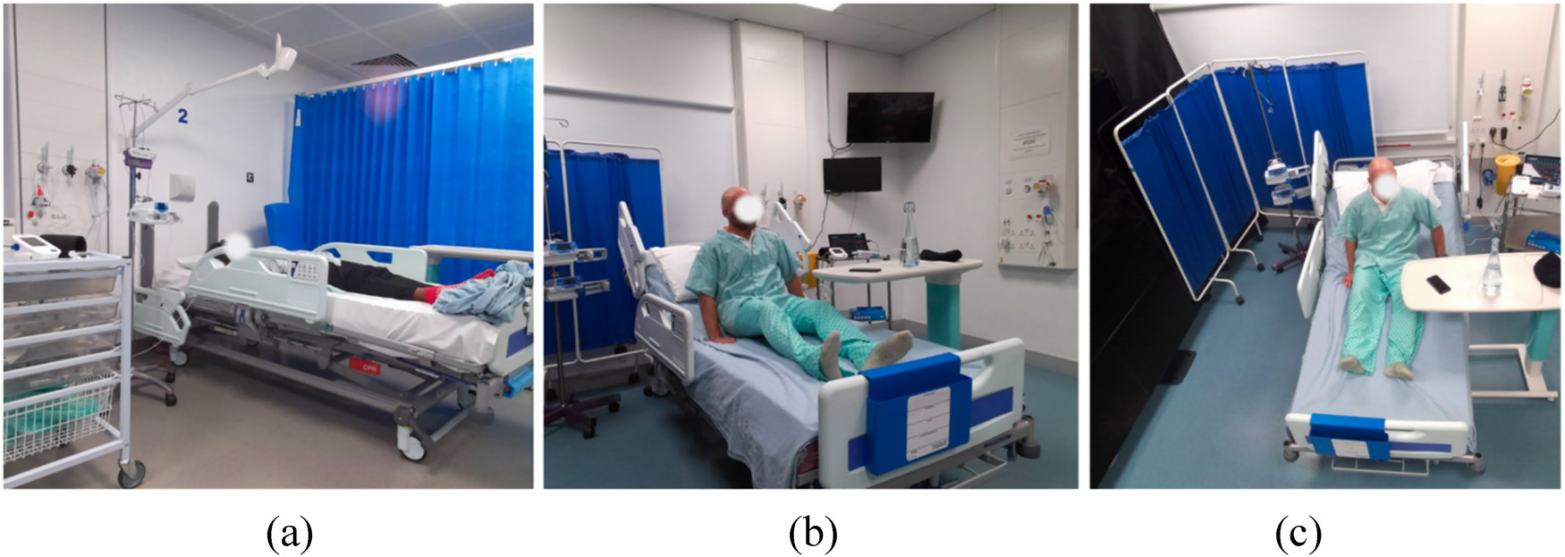
Camera viewpoints adopted in our simulated clinical environments: (a) Viewpoint 1, right side of the patient's bed; (b) Viewpoint 2 from the front-right of the bed; and (c) Viewpoint 3, above the bed.

We asked the participants to simulate common in-patient behaviours and activities, engaging in routine movements and interactions with clinical staff within discrete episodes. For each episode, we used two Microsoft Azure Kinect cameras to record the actions from three distinct viewpoints: (1) obliquely from left or right side of the patient's bed (Figure 7(a)), (2) obliquely in front of the bed (Figure 7(b)), and (3) above the bed (Figure 7(c)). We randomly sampled frames from videos shot from the three viewpoints. During preliminary exploratory evaluation, viewpoints 1 and 2 best exposed the vulnerabilities of standard object detection models and were therefore chosen for targeted synthesis (referred to as Scenario 1 and Scenario 2, respectively). Accordingly, we created two datasets for evaluating the detection models after tuning them on the synthetic datasets corresponding to the two selected viewpoints. The number and composition of validation samples were pragmatically determined. We first identified the video recordings where pre-trained models exhibited the most severe performance degradation – typically due to factors such as non-canonical viewpoints. From these recordings, we selectively sampled frames that captured representative and diverse body configurations. For viewpoint 1, we collected 224 frames with 226 manually annotated human instances and created a test dataset which will be referred to as ‘validation set 1’ in the following sections. For viewpoint 2, we collected 210 frames with 339 annotated human instances and created a corresponding test dataset which we will call ‘validation set 2’. Both collections contained frames of varying sizes, ranging from 480×640 to 1920×1080 , with annotations including both human bounding boxes and 2D poses. All participants’ bounding boxes and poses in these images were annotated according to standards proposed by Lin et al.,^36^ with the authors repeatedly verifying the process to ensure accuracy.

### Model training and performance evaluation

We selected five state-of-the-art object detection models (DINO, YOLOv6-L, YOLOv6-L6, and Salience-DETR with ResNet50 and FocalNet-L backbones) pre-trained on the MS-COCO instance dataset.^30–32,37–39^ These models vary in their core architecture (convolutional and transformer), layer connection arrangements (cascading, residual connection), attention mechanisms (FocalNet, self-attention), and capacity (25 to 250 million parameters).^38–41^

Specifically, DINO employs a transformer-based architecture with learnable query-based detection and de-noising training objectives. YOLOv6-L and YOLOv6-L6 represent efficient convolutional neural network (CNN)-based single-stage detectors, with YOLOv6-L using an anchor-free design with re-parameterised backbones, while YOLOv6-L6 extends this with enhanced multi-scale feature pyramids for higher-resolution processing. The Salience-DETR variants combine transformer-based detection heads (DETR) with different convolutional backbones: FocalNet provides hierarchical attention mechanisms for multi-scale feature extraction, while ResNet-50 offers standard convolutional feature representations. All models were initialised with COCO pre-trained weights before tuning with synthetic datasets.

Each model was tuned for up to 20 epochs on four datasets: dataset A, containing 10,800 randomly sampled images from the MS-COCO human keypoint dataset^36^; and three synthetic datasets each containing 10,800 samples adapted to three camera viewpoints. Dataset B contained images from an oblique viewpoint (a range spanning from −35° to 35° relative to the right side of the lying human body mesh, 0° indicates the bed's exact right side), dataset C from front right (15° to 35° to the left around the mesh, 0° indicates the exact front of the bed), and dataset D directly in front of the mesh. The learning rates for tuning all models were set at 0.1 of the learning rates used at their final pre-training stage. For example, the Salience-DETR models were trained with a batch size of 10 and a schedule using 1 × 10^−5^ at the final training stage^32^; their learning rates in our experiments were set to 1 × 10^−6^, also with a batch size of 10. However, for other models, due to hardware discrepancies, we set the base learning rate using the same strategy but scaled the actual learning rate according to the actual batch size (16) during training. No further hyper-parameter tuning was involved. For evaluation, we adopted the tuned checkpoints from the fifth epoch for all models, as overfitting became evident in later stages.

During training, we did not use any extra image augmentation than the models’ original image processing pipelines when training models on datasets B, C, and D. However, when training models on dataset A, image rotation was applied during the image pre-processing stage to mimic the scenes shown in Scenario 1 (Figure 7(a)), as most of the human instances are upright, which contrasts with the poses observed in Scenario 1. By applying rotations randomly sampled from 70° to 110° and −110° to −70°, most of the human instances were transformed to simulate the lying-down poses in side-view as frequently seen in Scenario 1. For human detection models, we evaluated several metrics including the precision and recall of human bounding boxes. For Scenario 1, we compared the training outcomes of datasets A, B, and D. Scenario 2 (Figure 7(b)) could not be simulated by simply transforming images in 2D, so we tested only the models trained on synthetic datasets C and D. For evaluating the performance of the overall pose estimation pipeline, we combined the tuned human detection models with state-of-the-art pose estimation models including HRNet – high-resolution CNN with unbiased data processing (UDP) and sub-pixel refinement (DARK), and ViTPose – Vision Transformer for hierarchical pose estimation.^42–45^ This selection enables systematic evaluation of synthetic retargeting across both established CNN-based approaches and emerging transformer architectures. All models used COCO pre-trained weights. We also tested the precision and recall of the segmented poses on the corresponding validation datasets. A summary of the datasets can be found in Table 1.

**Table 1.** A summary of the curated datasets.

Dataset	A	B	C	D	Validation 1	Validation 2
Image number	10,800	10,800	10,800	10,800	224	210
Whether targeted	Targeted for Scenario 1	Targeted for Scenario 1	Targeted for Scenario 2	Non-targeted	Targeted for Scenario 1	Targeted for Scenario 2
Train set size	10,000	10,000	10,000	10,000	0	0
Validation set size	800	800	800	800	0	0
Test set size	0	0	0	0	224	210

All tests were conducted using standard COCO metrics.^36^ For human detection, a predicted human bounding box is considered a true positive when it has an IoU value over a specific threshold with the ground-truth bounding box. The precision is defined as the portion of true positive bounding boxes among all the predicted ones. We calculated the mean AP and mean AR over multiple IoU thresholds (ranging from 0.5 to 0.95, with increments of 0.05), which comprehensively reflects the model's performance across different IoU requirements. Similar procedures were used for pose estimation evaluation, except that the IoU threshold was replaced by Object Keypoint Similarity (OKS).^36^

### Statistical analysis

Models were evaluated using an IoU-based scoring system. For each ground-truth person in the test sets, we:

1. calculated IoU between the predicted bounding box and ground-truth bounding box;
2. assigned a per-person score:

The 0.75 threshold ensures that only detections with substantial overlap are considered successful, maintaining high localisation quality appropriate for clinical applications. This scoring system simultaneously captures both detection sensitivity (whether a person was detected) and localisation accuracy (how precisely they were located).

We employed paired *t*-tests, Wilcoxon signed-rank tests, and bootstrap confidence intervals (CIs) for statistical analyses. The corrected statistical significance threshold was defined as *p* < 0.0011 for all hypothesis tests (*n* = 45). All statistical analyses were performed using Python 3 with scipy.stats and numpy libraries.

### Computational resources

The DINO and YOLOv6 models were trained using 8 NVIDIA Tesla P100 GPUs, the Salience-DETR models were trained on 4 NVIDIA Tesla V100 GPUs with a batch size of 10.^30–32^ The synthetic images were generated on a workstation running on Ubuntu 20.04 with an NVIDIA RTX2080Ti GPU and an AMD EPYC 7551P 32-Core Processor. The synthesis pipeline achieved an average generation time of 1.3 s per image (∼2770 images per hour) using batch size = 1 for SMPL-X generation. System RAM consumption was consistently maintained below 16 GB, while GPU VRAM consumption can vary significantly depending on batch size.

## Results

### Targeted synthesis

**Figure 8.**
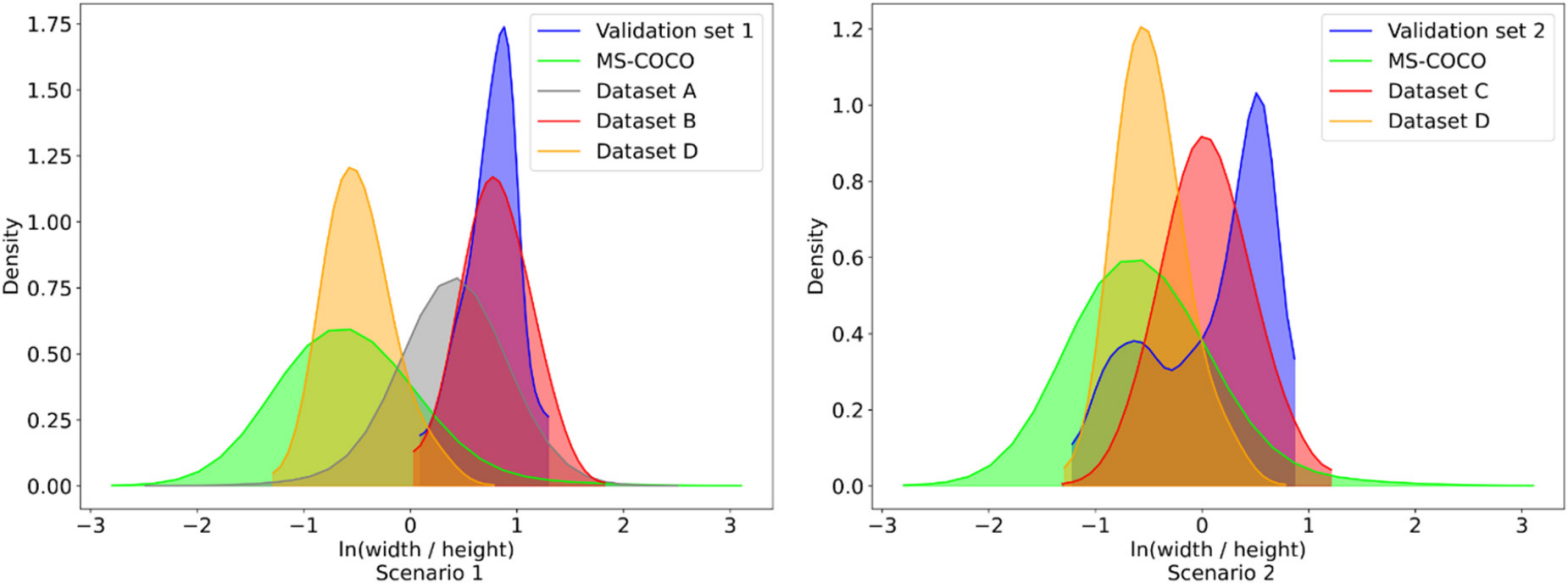
Probability density corresponding to each bounding box's ln(width/height) value in the datasets. Lower value indicates a more 'upright' pose, higher value indicates a more 'lying down' pose. A value of 0 means the bounding box appears as a square.

To convey the impact of the choice of camera viewpoint, Figure 8 shows the distribution of width and height ratios of captured human instances in all datasets. As shown in the left plot in Figure 8, the augmented COCO dataset (dataset A, in grey) is closer to the target distribution than the original dataset (MS-COCO, in lime green). Dataset B, however, in which the viewpoint is synthetically replicated in 3D is substantially closer. Although dataset D is closer to the target distribution than the MS-COCO instance dataset, the difference remains substantial. Nevertheless, the diversity of human appearances potentially contributed to the model's generalisability according to the results shown in later sections.

It can be seen from the right plot in Figure 8 that compared with the original MS-COCO instance dataset (in lime green), both datasets C (in red) and D (in orange) show a shift towards the target distribution in validation set 2, with C relatively better overlapping with the target than the other two datasets.

**Figure 9.**
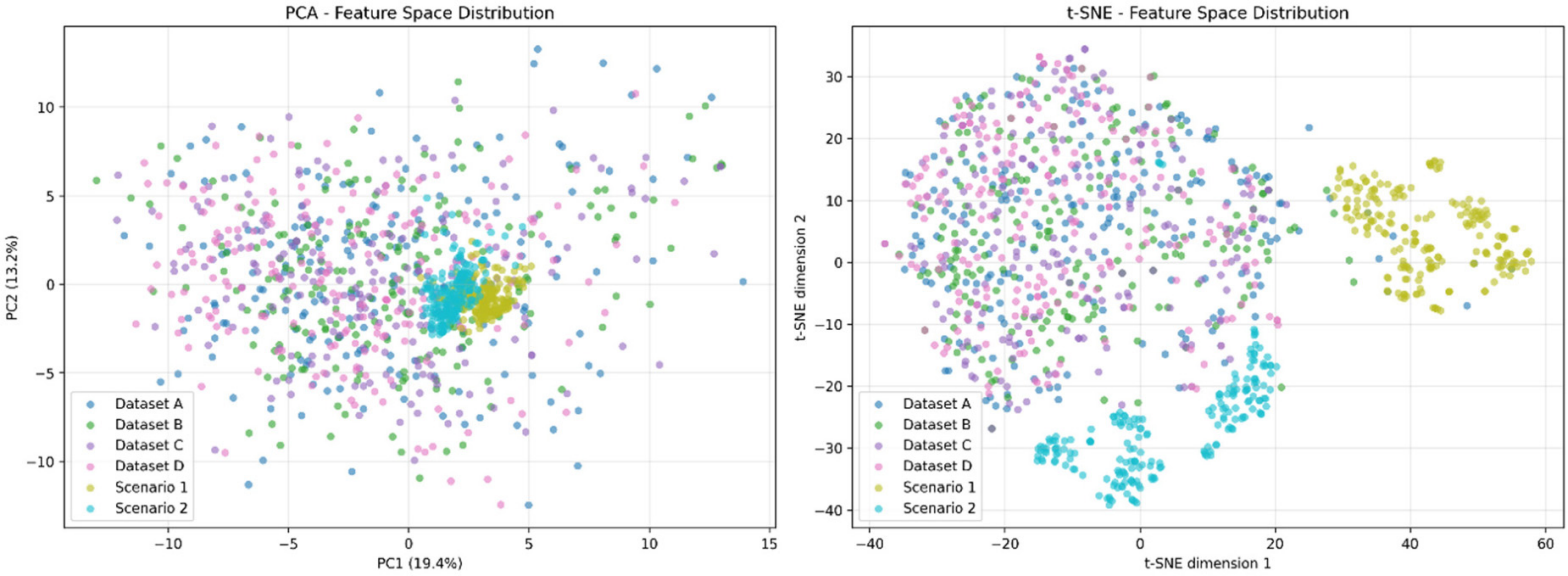
2D visualised PCA and t-SNE of VGG16-processed samples from the datasets. 2D: two-dimensional; PCA, principal component analysis; t-SNE, t-distributed stochastic neighbor embedding.

We additionally employed VGG16 to process all datasets mentioned above. We sampled 200 images from each dataset, extracted features through VGG16, and performed dimensionality reduction using principal component analysis (PCA) and t-distributed stochastic neighbor embedding (t-SNE) for visualisation. The results in Figure 9 revealed that, due to their environmental specificity, Scenarios 1 and 2 exhibited distinctive clustering in both PCA and t-SNE projections. While they showed some overlap with other data points in PCA space, they demonstrated more pronounced internal clustering. In t-SNE space, they were clearly separated from other data points. This indicates that environmental specificity (including acquisition equipment, background colours, and object occurrence frequencies) induces substantial feature shifts in computer vision models.

Simultaneously, we observed that our synthetic datasets (Datasets B, C, D) showed considerable overlap with the COCO-sampled dataset (Dataset A), suggesting that the generated data shares certain feature similarities with real-world data. This demonstrates the potential for reducing the domain gap.

### Human detection

Owing to the challenging viewpoints characteristic of clinical contexts, the models tended to predict bounding boxes with substantially lower confidence scores compared with ordinary scenes. We therefore de-noised inferred bounding boxes by thresholding their confidence scores at 0.001 and applied a separate confidence threshold of 0.2 for pose estimation tasks. Since the models frequently predicted multiple overlapping bounding boxes, we applied non-maximum suppression (NMS) with an IoU threshold at 0.65, a default value adopted from the MMPose code base to facilitate the subsequent pose estimation task.^46^

**Figure 10.**
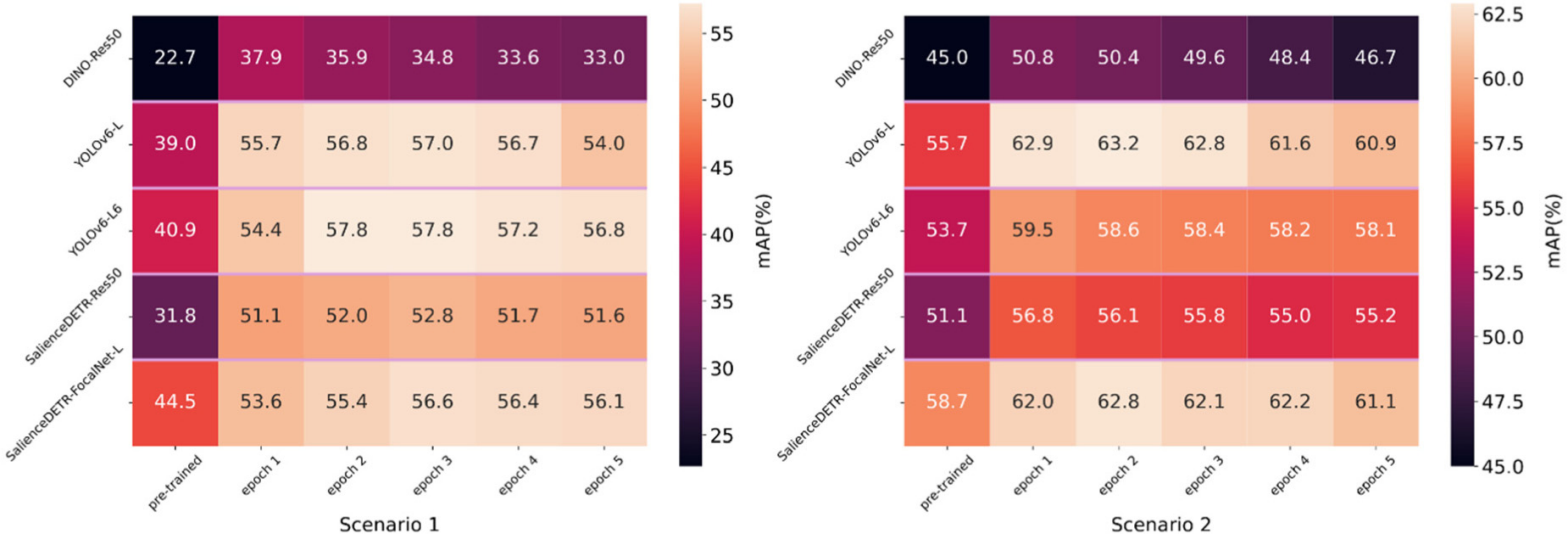
Human bounding box evaluation on validation set 1 (left) and validation set 2 (right) with models trained within five epochs (confidence score threshold = 0.001, NMS IoU threshold = 0.65). NMS: non-maximum suppression; IoU: intersection over union.

The results on the validation sets in Figure 10 show significant improvement within five epochs of training but progressive deterioration thereafter, a sign of overfitting. This is expected considering the reality gap and the much smaller scale of our synthetic dataset compared with publicly available datasets such as MS-COCO.^36^ For clinical applications, it is important that the training can improve the models at an early stage because there is usually limited computer hardware and human resources available for such tasks, and unlike evaluations on publicly available datasets, there are no clear standards for when to stop training due to the absence of a validation set under zero-shot or few-shot circumstances. To evaluate quick adaptation, we therefore compared the training outcomes after one training epoch in Tables 2a and b.

**Table 2.** Performance of human detection in two scenarios.

(a) Scenario 1. Human detection (bounding box), NMS IoU threshold = 0.65, on validation set 1								
	Confidence score threshold = 0.001				Confidence score threshold = 0.2			
Model	Before tuning	Dataset A	Dataset B	Dataset D	Before tuning	Dataset A	Dataset B	Dataset D
DINO-Res50	22.7|56.2	32.4|68.5	**37.9**|**64.2**	21.7|56.9	19.9|38.1	31.4|52.5	**35.2**|**46.4**	19.4|32.1
YOLOv6-L	39.0|62.2	45.7|67.5	**55.7**|**72.6**	48.8|67.6	34.7|45.1	42.9|55.4	**53.8**|**64.2**	45.8|55.5
YOLOv6-L6	40.9|62.7	34.2|60.9	**54.4**|**69.1**	47.9|67.4	37.4|46.7	31.3|42.5	**53.1**|**63.1**	46.0|56.9
Salience-DETR-Res50	31.8|57.9	44.7|67.6	**51.1**|**70.8**	33.8|63.3	31.0|49.4	43.8|60.8	**47.8**|**63.9**	32.5|51.9
Salience-DETR-FocalNet-L	44.5|64.4	**53.8**|**74.8**	53.6|72.2	46.5|67.3	43.8|59.3	**53.5**|**72.9**	53.3|69.6	45.4|60.1

(b) Scenario 2. Human detection (bounding box), NMS IoU threshold = 0.65, on validation set 2						
	Confidence score threshold = 0.001			Confidence score threshold = 0.2		
Model	Before tuning	Dataset C	Dataset D	Before tuning	Dataset C	Dataset D
DINO-Res50	45.0|65.0	**50.8**|**73.2**	47.1|72.9	22.7|54.7	**47.3**|**59.9**	44.7|57.6
YOLOv6-L	55.7|71.0	62.9|75.1	**63.0**|**75.3**	53.2|62.7	**61.6**|**69.9**	61.2|69.0
YOLOv6-L6	53.7|68.0	59.5|74.0	**60.4**|**74.6**	50.7|58.9	58.5|67.8	**58.6**|**67.7**
Salience-DETR-Res50	51.1|66.1	**56.8**|**72.0**	55.2|71.4	50.4|62.5	**55.6**|**68.2**	54.5|66.9
Salience-DETR-FocalNet-L	58.7|71.2	**62.0**|**75.3**	61.4|75.4	58.1|68.3	**61.3**|**70.0**	60.2|69.5

NMS: non-maximum suppression; IoU: intersection over union.

The results were measured using the models after one epoch of training. Results shown as AP|AR (average precision (%) | average recall (%)). Higher values indicate better performance. Best results are shown in bold.

For Scenario 1, we evaluated the models trained on Datasets A, B, and D on validation set 1. Comparing the performance of Datasets A and B in Table 2a, we observe that the targeted dataset can compete with and in most cases outperform the augmented real-world dataset (A), demonstrating the effectiveness of this approach in adapting pre-trained models to the target domain.

Comparing Datasets B and D, the non-targeted dataset (D) is clearly less effective. Comparing Datasets A and D, the results show that they are comparable, but both are less effective than Dataset B for training. The outcomes generally match the distribution differences shown in the left plot of Figure 8: Dataset B's histogram is the closest to the target domain.

For Scenario 2, we evaluated the models trained on Datasets C and D on validation set 2. In this scenario, the camera angles are less challenging than in Scenario 1. Both Datasets C and D brought improvements, but Dataset C demonstrated a minor advantage in most cases (Table 2b). Therefore, the effectiveness of targeted synthesis persists. Additionally, it matches the fact that compared to Scenario 1, Scenario 2 is less challenging according to the AP and AR measured using only pre-trained object detection models. Therefore, comparatively closer performance between Datasets C and D is expected.

### Pose estimation

Using the retargeted object detection model to compute human bounding boxes on the input images, we evaluated five state-of-the-art 2D pose estimation models on both validation sets, including the convolutional model (HRNet) and the transformer-based model (ViTPose). As shown in Table 3, the improvement in human detection contributes to higher precision in pose estimation. Although there was some marginal negative impact on ViTPose-base with a smaller capacity, the pose estimation models with larger capacity, such as HRNet and ViTPose-large, consistently benefited from increased robustness in human detection.^42,45^ This implies that the extra information provided by improved human bounding boxes might not substantially benefit less capable pose estimation models but can be well utilised by larger models, leading to significant improvements.

**Table 3.** Performance of pose estimation in two scenarios.

(a) Scenario 1: Human 2D pose estimation on validation set 1										
	HRNet-UDP		HRNet-DARK		ViTPose-base		ViTPose-large		ViTPose-huge	
Model	Before	After	Before	After	Before	After	Before	After	Before	After
DINO-Res50	60.9|77.5	**67.7**|**76.9**	62.5|78.8	**71.3**|**79.2**	59.9|75.8	**63.6**|**73.5**	67.4|82.0	**74.7**|**82.3**	68.6|84.0	**76.5**|**84.7**
YOLOv6-L	73.2|79.3	**77.7**|**82.4**	73.0|79.3	**79.9**|**84.1**	69.3|75.6	**72.6**|**77.7**	75.0|80.7	**82.1**|**86.0**	76.7|82.4	**84.5**|**88.3**
YOLOv6-L6	73.0|78.3	**77.2**|**82.0**	73.0|78.4	**79.2**|**83.5**	70.4|75.8	**73.8**|**78.7**	75.3|79.8	**81.7**|**85.5**	76.5|81.0	**83.6**|**87.4**
Salience-DETR-Res50	74.3|83.3	**75.4**|**82.1**	73.9|83.1	**78.2**|**84.8**	69.4|78.3	**72.0**|**78.9**	75.9|83.5	**80.4**|**86.1**	77.9|85.7	**83.4**|**88.6**
Salience-DETR-FocalNet-L	**78.1**|**84.5**	76.9|83.7	78.6|85.5	**79.0**|**85.3**	**75.1**|**82.0**	73.5|81.4	**81.6**|**88.3**	80.8|86.9	**83.6**|**89.9**	83.6|89.0

(b) Scenario 2: Human 2D pose estimation on validation set 2										
	HRNet-UDP		HRNet-DARK		ViTPose-base		ViTPose-large		ViTPose-huge	
Model	Before	After	Before	After	Before	After	Before	After	Before	After
DINO-Res50	69.0|85.9	**81.2**|**85.6**	71.7|86.3	**81.0**|**85.4**	67.7|85.8	**80.6**|**85.3**	70.1|88.9	**85.7**|**89.5**	73.5|90.5	**86.9**|**90.4**
YOLOv6-L	84.3|86.7	**84.8|87.8**	84.0|86.7	**85.2|88.0**	82.7|85.6	**83.6|86.6**	87.6|89.9	**89.6|91.7**	88.3|90.4	**90.8|93.0**
YOLOv6-L6	82.9|85.5	**84.6**|**88.1**	82.9|85.7	**84.9**|**88.4**	82.0|85.0	**83.7**|**87.3**	86.3|88.4	**88.5**|**91.2**	86.8|89.1	**90.1**|**92.6**
Salience-DETR-Res50	83.2|87.6	**84.3**|**88.8**	83.5|87.8	**84.6**|**89.0**	83.3|87.4	**83.5**|**88.3**	88.1|91.3	**89.5**|**92.9**	88.1|91.2	**90.2**|**93.6**
Salience-DETR-FocalNet-L	85.4|88.9	**85.9**|**89.**	85.8|89.0	**85.9**|**89.4**	**84.8**|**88.1**	84.5|88.4	89.4|92.2	**90.1**|**93.1**	90.4|93.0	**91.0**|**93.6**

2D: two-dimensional.

Results shown as AP|AR (average precision (%) | average recall (%)). Human bounding boxes provided by different detection models (settings identical to Table 2). Higher values indicate better performance.

### Statistical results

Statistical significance was assessed using both paired *t*-tests and Wilcoxon signed-rank tests, with all reported improvements showing concordant results across both methods. We conducted 45 tests in total for thorough statistical analyses across all datasets; therefore, the corrected *α* level was set to 0.0011 (Bonferroni correction). Due to the rectified scoring system mentioned in section ‘Statistical analysis’, the normality assumption does not hold, and therefore the Wilcoxon signed-rank test is considered the primary criterion (results shown in bold), while the paired *t*-test is considered supplementary.

Table 4 demonstrates the performance improvements achieved through targeted synthetic data in scenario 1. All models achieved improvements ranging from 10.7% to 19.4% (all *p* < 0.0011).

**Table 4.** IoU score improvements (averaged across instances) for human detection in Scenario 1, comparing pre-trained versus Dataset B-tuned models (confidence threshold = 0.2, NMS IoU threshold = 0.65).

Model architecture	Improvement (%)	95% confidence interval	p_t	p_w
DINO-Res50	+10.8	[5.5%, 15.9%]	<0.0001*	<0.0001*
YOLOv6-L	+19.4	[14.3%, 24.3%]	<0.0001*	<0.0001*
YOLOv6-L6	+15.7	[10.2%, 21.2%]	<0.0001*	<0.0001*
Salience-DETR-Res50	+15.7	[11.5%, 20.5%]	<0.0001*	<0.0001*
Salience-DETR-FocalNet-L	+10.7	[5.9%, 15.4%]	0.0001*	<0.0001*

NMS: non-maximum suppression; IoU: intersection over union.

Statistical significance was assessed using paired *t*-tests and Wilcoxon signed-rank tests. p_t: *p*-values of *t*-test; p_w: *p*-values of Wilcoxon signed-rank test. p_w is shown in bold as the primary criterion.

*Statistical significance after Bonferroni correction (*α* = 0.0011).

Combining the training outcomes in Table 2a with columns 2–4 (left to right) in Table 5, we observe that Dataset B most consistently produces meaningful improvements, while Dataset A also robustly improves model performance compared to Dataset D. Given that both Datasets A and B are targeted datasets specialised for Scenario 1, this demonstrates the superiority of targeted training in challenging contexts such as Scenario 1. Among the rightmost three columns of Table 5, the performance difference between Datasets B and D is the most significant, further emphasising the impact of targeted training.

**Table 5.** Statistical analyses (*p*-values) of performance differences between models trained with different datasets within the same model group (confidence threshold = 0.2, NMS IoU threshold = 0.65) in Scenario 1.

	Pre-trained vs. A		Pre-trained vs. B		Pre-trained vs. D		A vs. B		A vs. D		B vs. D	
Model architecture	p_t	p_w	p_t	p_w	p_t	p_w	p_t	p_w	p_t	p_w	p_t	p_w
DINO-Res50	<0.0001*	<0.0001*	<0.0001*	<0.0001*	0.0251	**0.6956**	<0.0001*	0.0004*	<0.0001*	<0.0001*	<0.0001*	<0.0001*
YOLOv6-L	<0.0001*	<0.0001*	<0.0001*	<0.0001*	<0.0001*	<0.0001*	<0.0001*	<0.0001*	0.4158	**0.0111**	<0.0001*	<0.0001*
YOLOv6-L6	0.0056	**0.1611**	<0.0001*	<0.0001*	<0.0001*	<0.0001*	<0.0001*	<0.0001*	<0.0001*	<0.0001*	0.0182	<0.0001*
Salience-DETR-Res50	<0.0001*	<0.0001*	<0.0001*	<0.0001*	0.0311	<0.0001*	0.0303	**0.0853**	0.0074	<0.0001*	<0.0001*	<0.0001*
Salience-DETR- FocalNet-L	<0.0001*	<0.0001*	<0.0001*	<0.0001*	0.2098	<0.0001*	0.0752	**0.0085**	<0.0001*	<0.0001*	0.0016	<0.0001*

NMS: non-maximum suppression; IoU: intersection over union.

p_t: *p*-values of *t*-test; p_w: *p*-values of Wilcoxon signed-rank test. p_w is shown in bold as the primary criterion.

*Statistical significance after Bonferroni correction (*α* = 0.0011).

**Table 6.** IoU score improvements (averaged across instances) for human detection in scenario 2, comparing pre-trained vs. Dataset C-tuned models (confidence threshold = 0.2, NMS IoU threshold = 0.65).

Model architecture	Improvement (%)	95% confidence interval	p_t	p_w
DINO-Res50	+7.3	[4.4%, 10.3%]	<0.0001*	<0.0001*
YOLOv6-L	+6.3	[3.6%, 9.6%]	<0.0001*	<0.0001*
YOLOv6-L6	+9.8	[7.0%, 12.9%]	<0.0001*	<0.0001*
Salience-DETR-Res50	+6.7	[3.9%, 9.6%]	<0.0001*	<0.0001*
Salience-DETR-FocalNet-L	+1.6	[−0.7%, 3.9%]	0.1555	<0.0001*

NMS: non-maximum suppression; IoU: intersection over union.

p_t: *p*-values of *t*-test; p_w: *p*-values of Wilcoxon signed-rank test. p_w is shown in bold as the primary criterion.

*Statistical significance after Bonferroni correction (*α* = 0.0011).

In Scenario 2, models trained with Dataset C demonstrated statistically significant improvements ranging from 6.3% to 9.8% for most architectures (all Wilcoxon signed-rank tests showed *p* < 0.0011) as shown in Table 6. YOLOv6-L6 achieved the largest gain (9.8%, 95% CI: 7.0–12.9%), while Salience-DETR with FocalNet backbone showed marginally significant improvement (1.6%, 95% CI: −0.7% to 3.9%, paired *t*-test *p* > 0.0011, Wilcoxon signed-rank test *p* < 0.0011). Notably, the more challenging deployment scenario (Scenario 1) benefited more from synthetic retargeting than the less constrained scenario (Scenario 2).

Combining the training outcomes in Table 2b with columns 2 and 3 in Table 7, we observe that Dataset C demonstrated more significant improvements in the Wilcoxon signed-rank test, while both datasets showed similar significance measures in the paired *t*-test. Compared to Scenario 1, the reduced performance difference between the targeted dataset (C) and non-targeted dataset (D) is expected, as Scenario 2 represents more common detection conditions that are better covered by the MS-COCO dataset used to pre-train the models (see Figure 8).

**Table 7.** Statistical analyses (*p*-values) of performance differences between models trained with different datasets within the same model group (confidence threshold = 0.2, NMS IoU threshold = 0.65) in Scenario 2.

	Pre-trained vs. C		Pre-trained vs. D		C vs. D	
Model architecture	p_t	p_w	p_t	p_w	p_t	p_w
DINO-Res50	<0.0001*	<0.0001*	0.0003*	<0.0001*	0.0740	**0.0031**
YOLOv6-L	<0.0001*	<0.0001*	<0.0001*	<0.0001*	0.8650	**0.0113**
YOLOv6-L6	<0.0001*	<0.0001*	<0.0001*	<0.0001*	0.6556	<0.0001*
Salience-DETR-Res50	<0.0001*	<0.0001*	0.0011*	<0.0001*	0.0735	**0.3384**
Salience-DETR-FocalNet-L	0.1555	<0.0001*	0.1992	<0.0001*	0.7237	**0.0715**

NMS: non-maximum suppression; IoU: intersection over union.

p_t: *p*-values of *t*-test; p_w: *p*-values of Wilcoxon signed-rank test. p_w is shown in bold as the primary criterion.

*Statistical significance after Bonferroni correction (*α* = 0.0011).

### Dataset ablation study

To evaluate the impact of scenario-specific targeting, we conducted ablation studies comparing Dataset D – generic synthetic data generated using the same procedural pipeline as Datasets B and C but without environmental matching – against our targeted configurations. Performance differences across all dataset configurations are presented in Tables 5 and 7.

The value of scenario-specific targeting varied substantially between deployment contexts. Dataset B achieved the largest improvements in Scenario 1 (10.8–19.4%, all *p* < 0.0011), while Dataset D showed markedly diminished effectiveness. This performance gap narrowed considerably in Scenario 2, where Dataset C remained superior in most cases, but Dataset D demonstrated comparable results across all tests. Additionally, Dataset D occasionally outperformed Dataset C for specific architectures in Scenario 2 (see Table 2b), indicating that generic augmentation can yield adequate benefits when environmental constraints are moderate.

## Discussion

Our findings suggest that targeted synthesis can enhance the performance of human detection models (as illustrated in the bottom panels of Figure 2), negating the need for specialised image augmentation and extensive hyper-parameter tuning. Our contribution may be summarised as follows:

1. We introduce synthetic retargeting as a mechanism for human detection and pose estimation in data and privacy-constrained settings such as healthcare.
2. We show that our method enables substantial improvements in detection performance compared with un-tuned state-of-the-art pre-trained models and is superior to conventional targeted augmentation such as specific geometric image transformations.
3. We show that synthetic retargeting can be highly efficient, achieving significant improvement within relatively few training steps compared to large-scale training or fine-tuning, while employing more user-friendly and streamlined training strategies.

Our study addresses a central problem in the modelling of human motion in clinical settings: satisfying the competing demands of high individual-level fidelity and context-level equity, and low dependence on labelled data within the specific environment. Reconciling these demands requires mechanisms both for introducing external inductive signals – from external data or prior knowledge – and for environment-specific tuning in the absence of labels. Here we emphasise these (hitherto neglected) requirements, and pursue one potential solution, based on targeted image synthesis, that can be used alone or in combination with complementary approaches such as large deep generative models of real-world video and human motion data. Below we consider the merits and de-merits of our approach, and outline a path towards delivering models of human motion, optimised for clinical settings, with the fidelity and equity the domain demands.

### Targeted image synthesis

We introduce an innovative data generation framework that synthesises virtual images tailored to specific scenarios. Compared with existing data generation methods, our framework arguably offers a more customisable and efficient solution. Previous studies have often faced limitations in dataset diversity and quality, required a substantial amount of manual design, and involved rigorously constrained object interactions.^10–14^ Our image synthesis approach minimises manual interference, presenting a lightweight, Python-based, entirely procedural human mesh generator with randomisable and flexible appearances and backgrounds, and can be used with any rendering engine.

### Human detection and pose estimation

We find significant improvements in human detection and pose estimation tasks when using our synthetic datasets tailored to specific camera angles. Our analysis shows that these synthetic datasets effectively simulate the camera angles encountered in clinical contexts, and cohere better with the target scenarios compared with public datasets. We adapted pre-trained object detection models for human detection to the target scenes with minimal tuning. The object detection models trained with these synthetic datasets demonstrate significant improvements in human detection tasks. Additionally, these models have shown considerable contributions to downstream pose estimation applications. As the front end of many machine vision tasks, improvements in object detection can effectively translate into increased overall success and accuracy of relevant applications. The substantial improvement in both human bounding box precision and overall pose estimation precision suggests that our data generation framework effectively addresses the challenge of model adaptation to difficult camera angles in clinical scenarios. This enhancement is crucial for applications requiring equity, high accuracy, and reliability without compromising patient privacy.

### Ablation study

The statistical analyses and ablation study in sections ‘Statistical results’ and ‘Dataset ablation study’ reveal a systematic relationship between scenario difficulty and the necessity of targeted synthetic data. Scenario 1 presents an uncommon deployment environment characterised by constrained viewing angles, narrow visual fields, and limited contextual information – conditions poorly represented in standard pre-training datasets such as MS-COCO. Under these demanding circumstances, generic augmentation proved insufficient for model adaptation and, in some instances, degraded performance (YOLOv6-L6, Dataset A in Table 2a). Conversely, Scenario 2's more conventional configuration – with broader coverage in pre-training data – posed less severe adaptation challenges, enabling both targeted and generic approaches to succeed.

This pattern suggests a practical principle: the incremental benefit of targeted synthetic data generation increases proportionally with the degree to which deployment scenarios deviate from standard training distributions. When deployment conditions are well-represented in pre-training data, generic augmentation may suffice; however, as scenarios become more constrained or specialised, targeted domain adaptation becomes essential for achieving robust performance gains.

### Limitations

Although our results demonstrate the effectiveness of synthetic retargeting for human detection and pose estimation in a clinical context, several limitations exist and suggest directions for future work.

#### Synthetic data realism and domain gap

The realism of procedurally generated synthetic data, though sufficient for our target application, leaves room for improvement. Our VGG16 feature analysis (Figure 9) revealed that synthetic datasets (B, C, D) exhibited considerable overlap with COCO-sampled data (Dataset A), indicating partial success in bridging the domain gap. However, clinical test scenarios (1 and 2) demonstrated distinctive clustering in both PCA and t-SNE projections, suggesting that environmental specificity – including acquisition equipment characteristics, background colours, and object occurrence frequencies – induces substantial feature shifts that synthetic retargeting only partially addresses. The feature space separation was particularly pronounced in t-SNE visualisation, where clinical scenarios remained clearly separated from both COCO and synthetic training data. This suggests that while synthetic retargeting reduces the domain gap, complete elimination remains challenging even with state-of-the-art rendering engines. Post-processing with appropriately configured deep generative models could potentially narrow this gap further by refining texture realism and environmental context matching.

#### Manual configuration and scalability

The targeted synthetic data generation employed here requires manual specification of scenario characteristics, including camera parameters, lighting conditions, environmental contexts, and specific patient positioning. This approach allows retargeting without the use of any data-driven approach: the key privacy constraint we seek to overcome. Nonetheless, this process may be automated through meta-learning or automated feature extraction from small, privacy-vetted samples of target deployment data for enhanced practical applicability. Additionally, determining the optimal volume and diversity of synthetic data remains empirical: our experiments used fixed dataset sizes, but the relationship between synthetic data volume, diversity, and downstream performance requires systematic investigation.

#### Representational bias

Despite procedural randomisation, synthetic humans rely on pre-trained SMPL-X models and fixed mesh templates, introducing potential biases. VGG16 analysis revealed recognisable feature overlapping between synthetic datasets, suggesting systematic patterns despite diversity efforts.

#### Architecture-dependent effectiveness

Performance gains varied substantially across architectures. Salience-DETR-FocalNet-L showed less significant improvement in Scenario 2 (1.6%, paired *t*-test *p* > 0.0011, Wilcoxon signed-rank test *p < *0.0001), while YOLOv6-L6 gained 9.8% (paired *t*-test *p* < 0.0001, Wilcoxon signed-rank test *p* < 0.0001). This suggests complex interactions between model capacity, inductive biases, and synthetic data characteristics that warrant further investigation.

### Future directions

Several promising directions emerge from the limitations. First, iterative retargeting within an adaptive framework – where synthetic data generation responds dynamically to model uncertainty during deployment – could address evolving scenario characteristics. Second, enhanced motion realism through rendering with physics-based simulation could further reduce domain gap between synthetic and real-world data. Third, extending the framework beyond human detection and pose estimation to related tasks (activity recognition, patient re-identification, multi-person tracking) would validate its broader applicability. Finally, systematic investigation of the relationship between synthetic data characteristics (volume, diversity, realism) and downstream performance across model architectures and clinical contexts would provide principled guidance for practical deployment. Despite the limitations, our results demonstrate that synthetic retargeting provides a viable path towards robust clinical pose estimation under real-world constraints, with particularly strong performance in challenging deployment scenarios where traditional approaches are constrained.

### Potential healthcare applications

The framework has broad healthcare applications wherever privacy constraints limit real data collection but computational resources are available for model adaptation. Our approach is particularly suited for controlled clinical environments including in-patient gait analysis (as demonstrated), physical therapy monitoring, fall risk assessment, and rehabilitation progress tracking. The key requirement is sufficient data volume for scenario-specific synthetic retargeting.

Deployment in out-patient facilities is feasible but presents practical challenges. Direct implementation requires technical expertise for synthetic data generation and model fine-tuning, which may exceed typical out-patient capabilities. A more scalable approach involves healthcare technology service providers offering solutions: conducting site assessments, generating customised synthetic datasets, and delivering deployment-ready models tailored to specific out-patient environments. This service model would enable broad adoption while centralising the technical complexity.

## Conclusion

The great expressivity of machine learning models rests on the premise of drawing intelligence from training data with minimal inductive bias. This pre-supposes the availability of data with reasonable coverage of the target domain. Such coverage is difficult to achieve in healthcare settings owing to the challenges – sometimes insuperable – of obtaining data in the applicable regime, especially under constraints of privacy and lack of labelling resources. The difficulty is further amplified by the necessity of achieving good performance not just across the population, but equitably across diverse sub-populations, and indeed at the level of the individual patient. This problem has only two plausible solutions. The first is to develop generative models with an overview of the entire domain of possible appearances, a challenging task yet to be accomplished and arguably infeasible without access to larger-scale and more diverse data than current infrastructure allows. The second, pursued here, is to develop procedural synthetics with sufficient flexibility to target inadequately represented cases, and to deploy systems that can fine-tune pre-trained models specific to each clinical scenario. If this process can be rapidly rendered, such fine-tuning can be tightly localised, down to specific patients and environments, ensuring both fidelity and equity of performance at low cost, both financially and computationally.

In conclusion, we introduce synthetic retargeting as a novel method for delivering robust pose estimation in healthcare and other settings where training data is limited, and demonstrate its efficacy and ready applicability in the domain.

## Abbreviations

AP: average precision
AR: average recall
CNN: convolutional neural network
IoU: intersection over union
NMS: non-maximum suppression
PCA: principal component analysis
t-SNE: t-distributed stochastic neighbor embedding
